# Prevalence of methicillin-resistant *Staphylococcus aureus* in dairy farms: A systematic review and meta-analysis

**DOI:** 10.3389/fvets.2022.947154

**Published:** 2022-12-06

**Authors:** Shrijana Khanal, Sukolrat Boonyayatra, Nattakarn Awaiwanont

**Affiliations:** ^1^Faculty of Veterinary Medicine, Veterinary Public Health Centre for Asia Pacific, Chiang Mai University, Chiang Mai, Thailand; ^2^Department of Food Animal Clinic, Faculty of Veterinary Medicine, Chiang Mai University, Chiang Mai, Thailand; ^3^Faculty of Veterinary Medicine, Center of Excellence in Veterinary Public Health, Chiang Mai University, Chiang Mai, Thailand

**Keywords:** methicillin-resistant *Staphylococcus aureus*, MRSA, meta-analysis, systematic review, dairy cattle farm

## Abstract

Methicillin-resistant *Staphylococcus aureus* (MRSA) is an opportunistic bacterium that causes many human and animal infections worldwide. MRSA infections are classified as priority infections owing to their high morbidity and mortality, with a significant risk of zoonotic transmission. This study aimed to determine the pooled prevalence of MRSA in dairy cattle farms and its heterogeneity. Relevant studies were retrieved from three databases: PubMed, Web of Science, and Scopus. The pooled prevalence of MRSA in dairy farms was estimated using a random-effects model. Subgroup and meta-regression analyses were used to assess the probable sources of heterogeneity. Sensitivity and publication bias analyses were also performed. A total of 94 articles were eligible for inclusion in this meta-analysis. The pooled prevalence of MRSA was estimated to be 3.81% [95% confidence interval (95% CI) = 2.61–5.20] with significantly high heterogeneity (*I*^2^ = 96.6%, *p* = 0.00). For the subgroup analysis among continents, the prevalence was highest in Asia (4.89%; 95% CI = 2.88–7.35) and lowest in South America (1.33%, 95% CI = 0.00–5.49). As for the year of publication, MRSA prevalence was highest in reports published from 2015 to 2018 (4.36%, 95% CI = 2.41–6.80) and lowest in reports published before 2015 (2.65%, 95% CI = 0.75–5.52). As for sample type, the prevalence of MRSA in cattle milk (3.91%, 95% CI = 2.64–5.39) was higher than that in other sample types (1.19%, 95% CI = 0.05–3.24). These three factors were not significantly associated with the pooled prevalence of MRSA (*p* > 0.05). Therefore, the findings of this study indicate that the prevalence of MRSA has been minimal and consistent in dairy cattle farms over time.

## Introduction

*Staphylococcus aureus* is a commensal bacterium that can be found on the skin, mucous membranes, and upper respiratory tracts of both animals and humans (1). However, it can be an opportunistic pathogen that causes various infectious illnesses in humans and animals (2). *S. aureus* is associated with many human disorders, from skin and soft tissue infections to life-threatening septicemia (3). In veterinary medicine, it is a common cause of bovine mastitis in dairy cattle, resulting in high economic losses worldwide (4).

Methicillin-resistant *S. aureus* (MRSA) was first documented in 1961 (5). MRSA strains were phenotypically identified using cefoxitin and oxacillin susceptibility assays (6). The gold standard for detecting MRSA is through the detection of the *mecA* gene, which encodes a protein called PBP2a, that has a poor affinity for β-lactam drugs, resulting in resistance to methicillin (7, 8). According to the recorded data, methicillin resistance has been identified in 50–70% of *S. aureus* strains isolated from the hospital environment, causing ~100,000 infections in the United States each year, with a 20% mortality rate (9). In 1972, MRSA was first reported in domestic animals as a pathogen causing bovine mastitis in dairy cattle in Belgium (10). Since then, various studies reported the zoonotic transmission of MRSA from livestock, especially pigs, poultry, and cattle, to farm workers and exposed people, which has been known as livestock-associated MRSA (LA-MRSA) (11–13). The majority of LA-MRSA isolates lack toxins such as PVL and enterotoxins (14) and are reported to have multiple antimicrobial resistance (15).

In the past two decades, numerous studies have reported different prevalence rates of MRSA on dairy cattle farms in different regions. These variations might be associated with isolation protocols, farm management systems, sample sizes, sample sources, and other factors (16). Most studies have detected MRSA in bovine mastitis cases. However, several studies have demonstrated the presence of MRSA in raw milk, farm workers, and dairy cattle farms, indicating the possible risk of MRSA transmission within dairy cattle farms and across the dairy supply chain to the general public (17–19). Hence, the objective of this study was to estimate the global prevalence of MRSA isolated from various sample sources in dairy cattle farms through a systematic review and meta-analysis of published articles.

## Materials and methods

### Search strategies

The Preferred Reporting Items for Systematic Review and Meta-analysis (PRISMA) guidelines were adopted for this study. Relevant studies published until 31 December 2021 were retrieved from three online databases: PubMed, Scopus, and Web of Science. The search was limited to original articles published in English. The keywords used for searching the relevant studies were “MRSA” OR “Methicillin-resistant *Staphylococcus aureus*” AND “dairy cattle” OR “dairy cow.”

### Inclusion and exclusion criteria

All original publications reporting the prevalence of MRSA, as determined by the detection of *mecA* and/or *mecC* genes, in dairy cattle farms were considered for analysis. The inclusion criteria were observational, cross-sectional, and case–control studies that determined the prevalence of MRSA from any sample source in dairy cattle farms. Studies were excluded from the analysis if they were (1) review articles, (2) experimental studies, (3) not written in English, (4) lack of a clear report on the prevalence of MRSA from any sample sources in dairy cattle farms, (5) lack of clear sample size, (6) performed on archived isolates, (7) no full text available, and (8) studies that used only phenotypic tests to detect MRSA. The titles and abstracts of the retrieved studies were evaluated for eligibility. After title and abstract screening, the full text of each article was thoroughly reviewed for inclusion. Two authors, SK and SB, independently performed study screening and selection. Disagreements were resolved through discussion.

### Data extraction

Two authors individually extracted data from all included studies. Discrepancies between the data obtained by these two authors were discussed with a third author for consensus to avoid bias. The extracted data included (1) the name of the author and year of publication, (2) the continent where the study was conducted, (3) sample size, (4) sources of samples, (5) the number of *S. aureus* isolates, (6) the number of MRSA isolates, and (7) the detection method used.

### Study quality assessment

The quality assessment criteria derived from Ding et al. (20) were used to evaluate the quality of the included studies. The checklist for determining the quality of studies consisted of these five questions: (1) Was the research objective clearly stated? (2) Was the sampling method described? (3) Was the study period and location clearly stated? (4) Were the examination methods and procedures for MRSA detection described clearly? (5) Were the samples clearly classified into different subgroups? The answers to each question were scored as “2” for yes, “0” for no, or “1” for unsure. A summation of the scores from all five questions was calculated, and the overall quality of each study was evaluated.

### Statistical analysis

Meta-analysis was performed using the R package meta in the statistics software R (21, 22). The prevalence of MRSA in dairy cattle farms was determined by dividing the number of MRSA isolates by the total sample size. Because several studies reported zero prevalence of MRSA, Freeman–Tukey double arcsine transformation was performed for all raw proportions before conducting the meta-analyses to avoid excluding these studies (23). The classic meta-analysis model utilizing logit-transformed proportions and the corresponding standard errors in the inverse variance method was used to pool studies (24). Back-transformation of all estimated pooled prevalence was performed for ease of interpretation.

A random-effects model was used to estimate the overall pooled prevalence of MRSA in dairy cattle farms, together with its 95% confidence interval (95% CI). Cochran's *Q*-test was used to determine the heterogeneity of the pooled prevalence. Furthermore, the *I*^2^ statistic was used to characterize the degree of heterogeneity across studies, with values of 25, 50, and 75% indicating low, medium, and high heterogeneity, respectively (25).

The subgroups in each study were used as the unit of analysis for all subgroup meta-analyses. Subgroup analyses were performed to investigate the heterogeneity between three variables: year of publication, continent, and sample type. The year of publication for each study was categorized into three groups consisting of “before 2015,” “2015–2018,” and “after 2018.” Each study was classified into five continents: “Asia,” “Africa,” “Europe,” “South America,” and “North America.” Sample type, referring to the sources of samples, was analyzed as two subgroups: “cattle milk” and “others.” The “cattle milk” category included quarter milk, composite milk, bulk tank milk, and milk from clinical and subclinical mastitis cases. Other sources of samples, such as cattle nasal swabs, human samples, and environmental samples collected from dairy cattle farms, were included in the “others” category.

Meta-regression analyses were performed to investigate the significance of the between-study heterogeneity associated with three independent variables: year of publication, continent, and sample type. Levels within each independent variable were similar to those described for the subgroup meta-analyses. A univariate meta-regression model was created to determine the association between each independent variable and the prevalence of MRSA in dairy cattle farms. Furthermore, variables with *p* ≤ 0.25 in the univariable meta-regression analysis were introduced to the random-effects multivariable meta-regression model.

Publication bias was examined using a funnel plot and Egger's test, where a *p* < 0.05 indicates statistical significance (26). The robustness of the results was evaluated using two sensitivity analyses. The first is a comparison of the results obtained from the random-effects and fixed-effects models. In addition, a leave-one-out meta-analysis was performed to evaluate whether any single study affected the results.

## Results

### Search results and study selection

**Figure 1:**
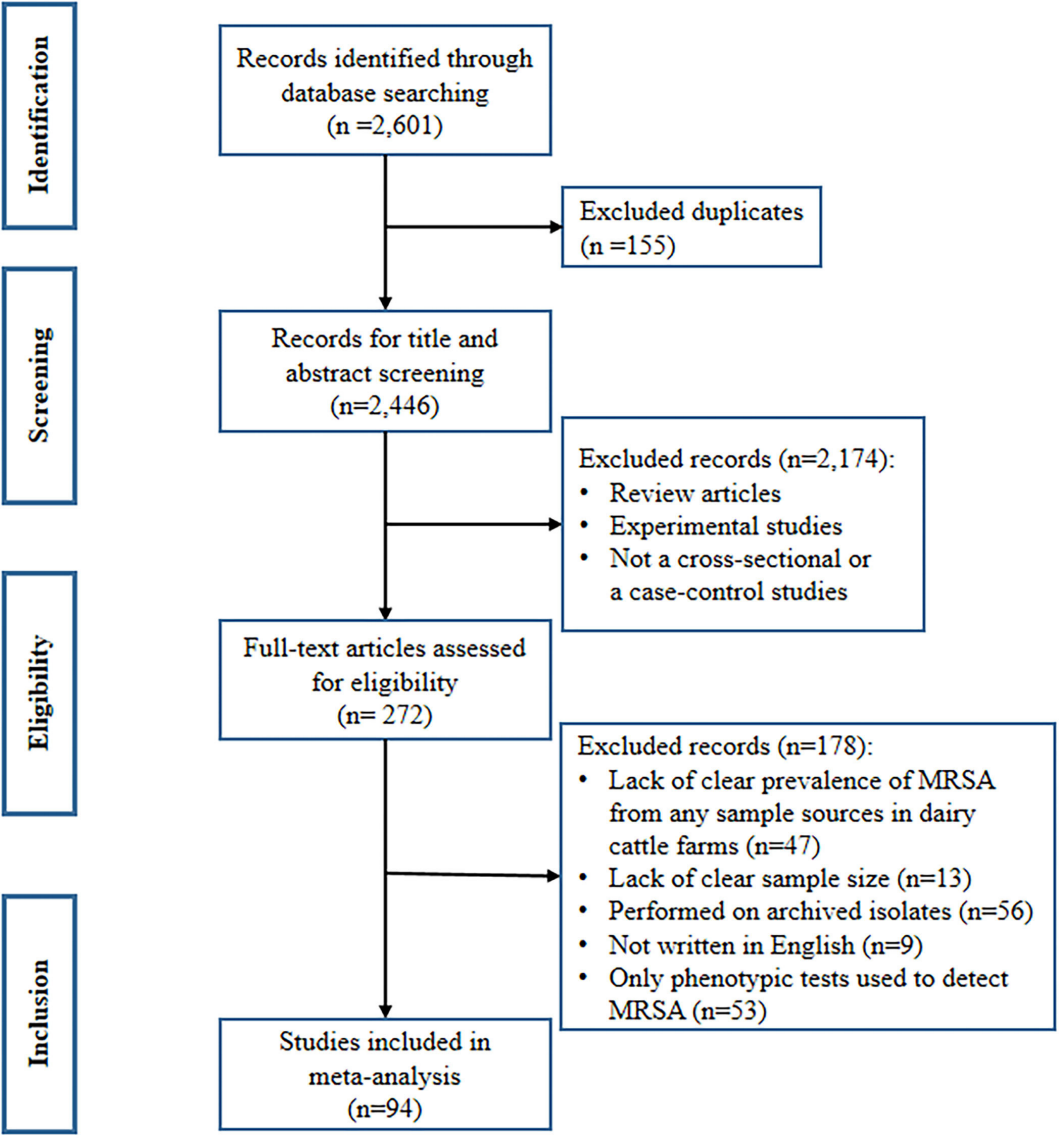
PRISMA flow diagram describing the selection process of the included studies.

A total of 2,601 records were identified from the three databases searched. These records consisted of 69 from PubMed, 2,446 from Scopus, and 86 from Web of Science. Of these, 155 records were duplicates and were removed before screening the titles and abstracts. After the screening process, 272 articles were included in the full-text review for inclusion criteria. Finally, 94 studies met the inclusion criteria and were included in the meta-analysis (16, 27–118). The remaining 178 articles were excluded for the following reasons: lack of clear prevalence of MRSA from any sample sources in dairy cattle farms (*n* = 47), lack of clear sample size (*n* = 13), archived isolates (*n* = 56), not written in English (*n* = 9), and studies that used only phenotypic tests to detect MRSA (*n* = 53), as shown in Figure 1.

### Characteristics of the included studies

The 94 studies considered in this review were published between 2003 and 2021, with the majority published after 2018 (*n* = 38). These studies reported the prevalence of MRSA in 30 countries across five continents. Most studies were conducted in Asia (*n* = 43), followed by Africa (*n* = 20) and Europe (*n* = 20). The majority of studies reported MRSA detection in milk samples (*n* = 90), whereas only 22 studies reported the presence of MRSA from other sample types. The mean ± standard deviation of quality scores of all included studies was 7.91 ± 1.62, with a range from 4 to 10. The characteristics of the selected studies are shown in Supplementary Table 1.

### Overall pooled prevalence of MRSA in dairy cattle farms

**Figure 2:**
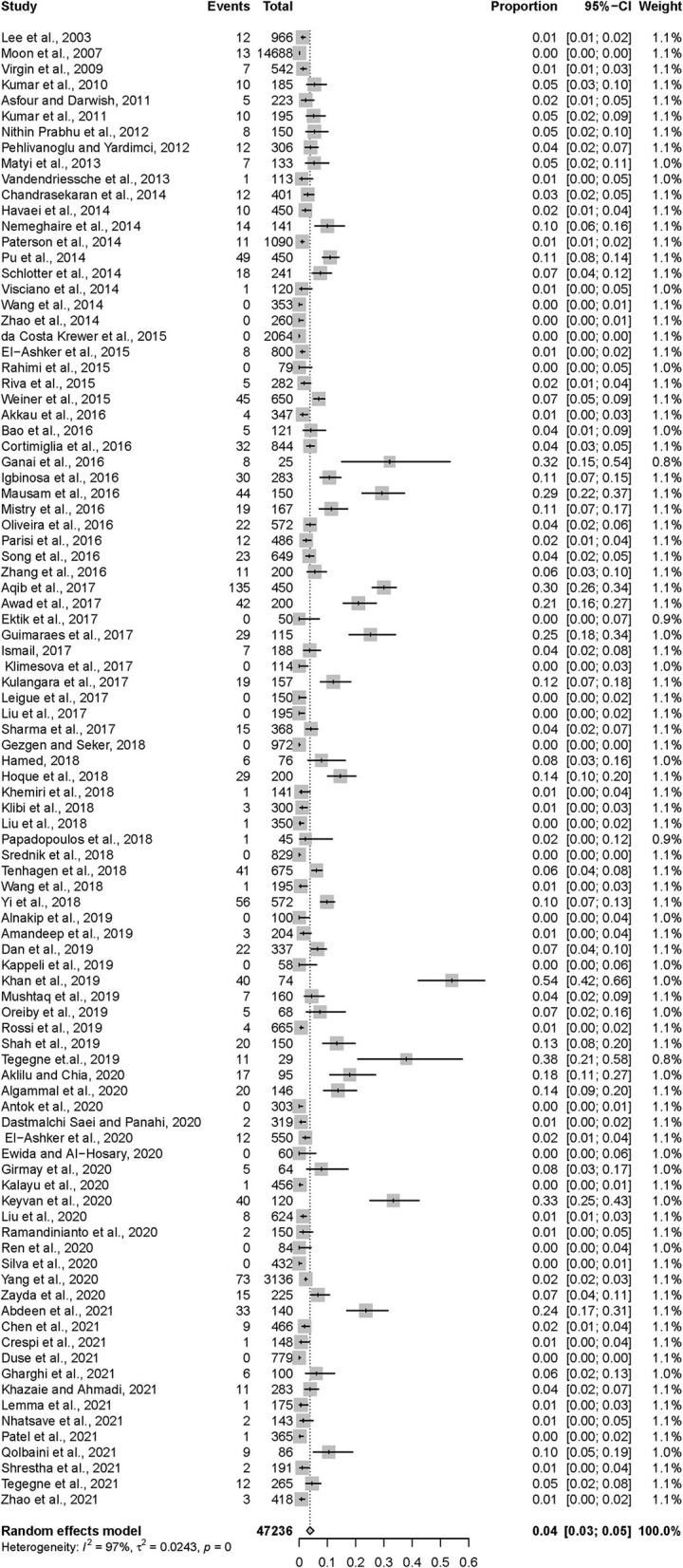
Forest plot demonstrating the pooled prevalence of MRSA in dairy cattle farms and its 95% confidence interval estimated by a random-effects model.

After data extraction, a total of 1,251 MRSA strains isolated from 47,236 samples collected from dairy cattle farms worldwide were included in the meta-analysis. As estimated from the random-effects model, the overall pooled prevalence of MRSA in dairy cattle farms was 3.81% (95% CI = 2.61–5.20), with high heterogeneity (*Q* = 2773.64; *I*^2^ = 96.6%; *p* = 0.00). These data are shown in Table 1 and Figure 2.

**Table 1:** Meta-analysis of methicillin-resistant *Staphylococcus aureus* (MRSA) prevalence from dairy cattle farms.

**Subgroups**	**No. of studies or subgroups**	**MRSA prevalence (%)**		**Heterogeneity**			***p*-values for subgroup differences**
		**Estimate**	**95% CI**	**Q**	***p***	***I^2^***	
Overall	94	3.81	2.61–5.20	2,773.64	0	96.6%	
Publication year							0.558
Before 2015	19	2.65	0.75–5.52	458.14	< 0.01	96.1%	
2015 to 2018	37	4.36	2.41–6.80	1,245.34	< 0.01	97.1%	
After 2018	38	3.94	2.10–6.28	675.93	< 0.01	94.5%	
Continent							0.307
Africa	20	3.92	1.79–6.76	303.18	< 0.01	93.7%	
Asia	43	4.89	2.88–7.35	1,727.65	0	97.6%	
Europe	20	3.19	0.99–6.38	392.32	< 0.01	95.2%	
North America	3	1.61	0.02–5.05	12.12	< 0.01	83.5%	
South America	8	1.33	0.00–5.49	176.50	< 0.01	96.0%	
Sample type							0.318
Cattle milk	90	3.91	2.64–5.39	2,692.99	0	96.7%	
Others	22	1.19	0.05–3.24	102.21	< 0.01	79.5%	

CI, confidence interval; Q, Cochran's Q-test for heterogeneity; I^2^, I^2^ statistic estimating the degree of heterogeneity across studies.

### Subgroup analysis and meta-regression analysis

The pooled prevalence of MRSA and the 95% CI for different subgroups of the year of publication, continent, and sample type are shown in Table 1. High heterogeneity was observed among all the tested subgroups. However, no statistically significant differences were detected between these subgroups. According to the year of publication, no significant trend in MRSA prevalence was observed, but the highest prevalence was observed among studies published between 2015 and 2018 (4.36%, 95% CI = 2.41–6.80). The pooled prevalence of MRSA in Asia appeared to be highest (4.89%, 95% CI = 2.88–7.35), followed by Africa (3.92%, 95% CI = 1.79–6.76) and Europe (3.19%, 95% CI = 0.99–6.38). The estimated prevalence of MRSA was lowest in South America (1.33%, 95% CI = 0.00–5.49). The pooled prevalence of MRSA in cattle milk (3.91%, 95% CI = 2.64–5.39) was higher than, but not statistically significantly different from, those in other sample types from dairy cattle farms (1.19%, 95% CI = 0.05–3.24). When the meta-regression models were analyzed for all three variables, no significant variable was associated with the heterogeneity of the overall pooled prevalence of MRSA in dairy cattle farms (Supplementary Tables 2, 3).

### Publication bias and sensitivity analysis

**Figure 3:**
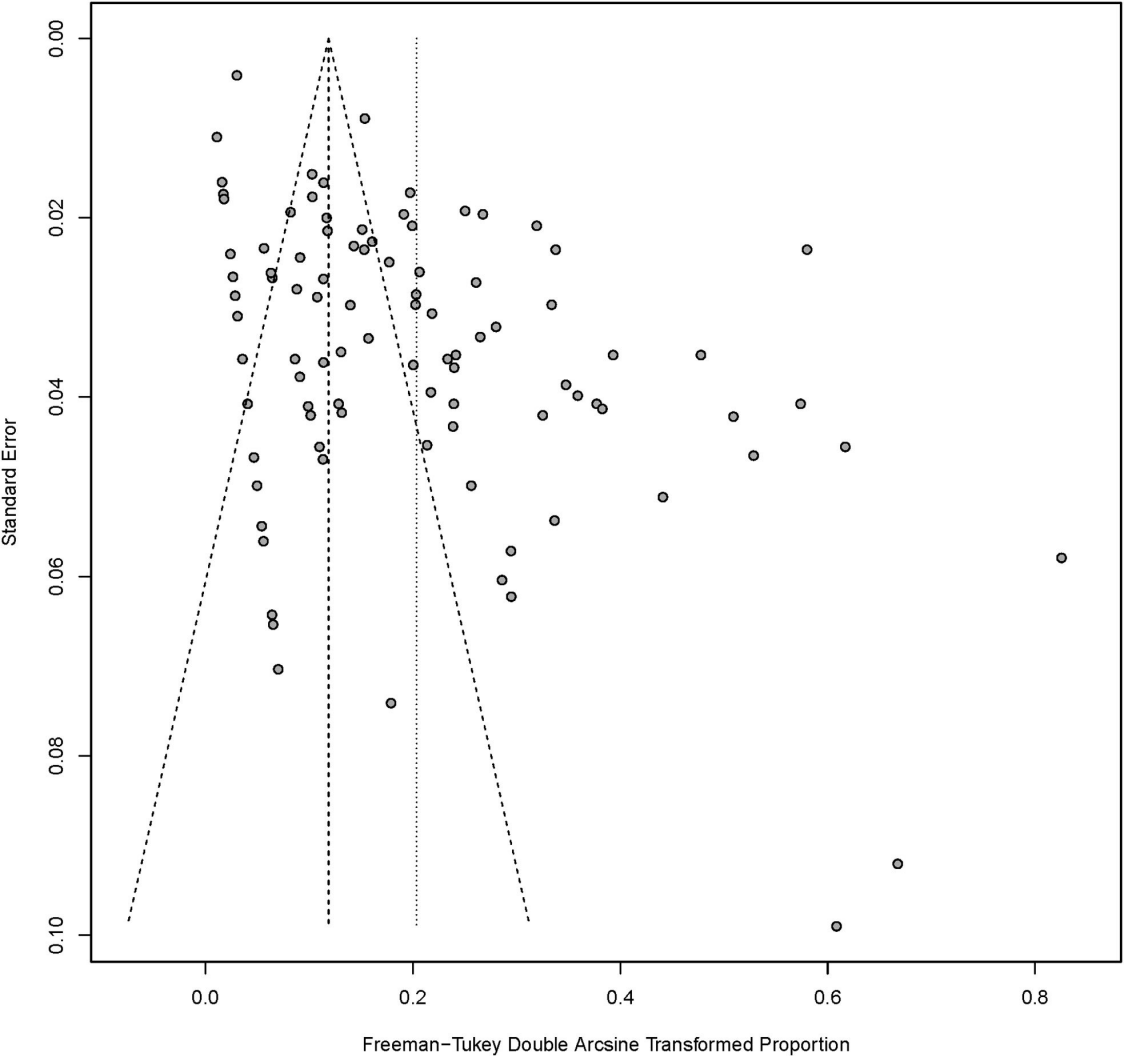
Funnel plot of data from all included studies examining the publication bias.

The funnel plot created from the data obtained from the selected studies demonstrated asymmetry of distribution, as shown in Figure 3, indicating a publication bias among the selected studies. To investigate the sources of funnel plot asymmetry, the results from Egger's test showed a statistically significant coefficient bias (5.30 ± 0.77, *p* < 0.0001), revealing evidence of small-study effects. Furthermore, sensitivity analysis was performed to assess the robustness of the models used to estimate the pooled prevalence of MRSA. The overall pooled prevalence of MRSA in dairy cattle farms using a fixed-effects model was much lower than that using a random-effects model, as shown in Table 2. In addition, a leave-one-out meta-analysis was performed to investigate the impact of each study on the pooled prevalence of MRSA in dairy cattle farms. Removing the studies with the lowest or highest prevalence did not significantly influence the overall pooled prevalence of MRSA in dairy cattle farms, as shown in Table 2.

**Table 2:** Sensitivity analysis to determine the robustness of the results obtained from the models used.

**Categories**	**No. of studies or subgroups**	**Prevalence (%)**	
		**Estimate**	**95% confidence interval**
Model
Fixed effects	94	1.12	1.01–1.22
Random effects	94	3.81	2.61–5.20
Leave-one-out analysis
The lowest prevalence^a^	93	3.55	2.47–4.80
The highest prevalence^b^	93	3.89	2.68–5.30

^a^The study by Khan et al. (86) was excluded from the meta-analysis.

^b^The study by da Costa Krewer et al. (46) was excluded from the meta-analysis.

## Discussion

The current study revealed that the global prevalence of MRSA isolated from various sample sources in dairy cattle farms, using a random-effects meta-analysis model, was 3.81%. Recently, Zaatout and Hezil (119) reported the global prevalence of MRSA isolated from bovine mastitis cases using a meta-analysis. Their reported prevalence was 4.30%, which was similar to our findings. The small variation in the estimated prevalence could be due to the fact that the current study included data from various sample types presented in dairy cattle farms, while the study by Zaatout and Hezil only selected reports from MRSA in the milk of clinical and subclinical bovine mastitis cases. We included data from a broad range of sample types to demonstrate the overall pooled prevalence of MRSA in dairy cattle farms, which can be used to determine the risk of MRSA transmission and contamination between cattle, humans, and the environment within the farms, and between the farms and other population at risk, especially the dairy consumers.

Subgroup analyses were carried out depending on the year of publication, continent, and sample type. We observed that the number of selected articles published before 2015 was limited (19/94). Increased numbers of studies were observed from 2015 to 2018 (37/94) and after 2018 (38/94). The highest pooled prevalence of MRSA was observed from 2015 to 2018 (4.36%) but not statistically different from that before 2015 and after 2018. In contrast, a recent meta-analysis on MRSA associated with bovine mastitis reported a significantly increasing trend in prevalence by the year of publication and suggested that it might be influenced by the advancements in the detection methods used (119). This contrast can be explained by the difference in included studies, the different categorization used to create levels for the subgroup analysis of the publication year, and the different sample types to be included in both studies. All of these differences might be resulted in narrower CIs of the reported prevalence of each level of year of publication in the previous study, compared to those of reported prevalence in the current study. The narrower CIs could be potentially associated with the statistical significance observed in the previous study. The changes in prevalence emphasize the importance of monitoring MRSA in dairy cattle farms to assess the progress or success of any implemented antimicrobial resistance control program.

Although we could not demonstrate a statistically significant difference in pooled prevalence among subgroups, our results showed a substantially higher prevalence of MRSA in dairy cattle farms in Asia (4.89%) than in South America (1.33%). The milk production and dairy animal population in Asia have been increasing (120). During the period from 2010 to 2020, cow milk production in Asia increased up to 4.2%, which was the highest growth compared to other regions of the world (121). This could be associated with the high number of research studies investigating the presence of any zoonotic pathogens, especially MRSA, in dairy cattle farms. The limited number of publications in South and North America can potentially lead to an underestimation of the prevalence of MRSA in dairy cattle farms in these regions and should be noted. Moreover, the higher prevalence of MRSA in Asia compared to other regions might be due to the high consumption of antimicrobial agents in food animals (122), which could be related to the increased dairy cattle population in this region and the available antimicrobial agents used in the region as they have a different selective pressure on MRSA. This phenomenon can also be attributed to the unethical use of antibiotics, especially in developing countries, where drugs are administered on the spur of the moment and without veterinarian monitoring (123). Another concern is poor farm sanitation and water management, both of which can facilitate MRSA transmission from animals to humans and vice versa and the development of antimicrobial resistance (124).

In addition to cattle milk, MRSA has been isolated from farm workers, farm environments, and other cattle organs. The pooled prevalence of MRSA in milk samples was lower than that in other sample sources from dairy cattle farms. Most milk samples reported in the selected studies were quarter milk samples collected aseptically; therefore, MRSA detected in cattle milk is generally a representative pathogenic strain of MRSA associated with intramammary infection and/or mastitis in cattle. In contrast, MRSA isolated from other sample sources could be either pathogenic or non-pathogenic strains, or a mix of both. Our findings suggest that MRSA is higher prevalent among bovine mastitis-causing *S. aureus* than other pathogenic or non-pathogenic *S. aureus* found in other sources in dairy cattle farms. However, the difference in the prevalence of MRSA isolated from these two sample types was not significant. Moreover, MRSA transmission among cattle, humans, and the environment cannot be ruled out. Therefore, MRSA monitoring and prudent antimicrobial use in dairy cattle farms should be regularly implemented.

Regarding univariable and multivariable meta-regression, there was no significant association between the overall pooled prevalence of MRSA and any variable, suggesting that the source of heterogeneity could not be explained by the year of publication, continent, or sample type. It suggests that the heterogeneity of reported prevalence among included publications might be associated with other factors, such as the method of isolation, the sampling and sample handling procedure, and the history of MRSA infection or transmission in the farms. However, the information regarding those factors was not equally and well-explained in most of the included publications. Therefore, they were not extracted during the systematic review and included in the meta-analysis. A further study with a more specific hypothesis using different search strategies and inclusion and exclusion criteria should be performed to investigate the source of heterogeneity of the prevalence of MRSA in dairy cattle farms.

Analysis of publication bias performed using the funnel plot and Egger's test revealed the bias of publications with small-study effects. Small-study effects are generally defined as a phenomenon in which studies with smaller sample size show different, and often larger, effects than studies with a larger sample size. This phenomenon can be due to the publication bias, when small studies reporting larger effects are more likely to be published compared to those reporting smaller effects. A funnel plot, showing the reported effects from small studies which are usually associated with high standard errors and large studies which are usually associated with low standard errors, can be used to illustrate the publication bias. According to the present study, the funnel plot clearly shows that small studies reporting low prevalence are missing which is illustrated as an area without any dots in the bottom left corner of the plot. Even though the small-study effect is a potential limitation of this study, all included publications were of fair to high quality. Moreover, using a sensitivity analysis, we showed that our meta-analysis was robust and stable. Other study limitations should also be concerned. First, only articles that were written in English were included. Second, the included studies were obtained from only three distinct databases. Third, the year of publication of several studies was not identical to the year of MRSA isolation, which may have influenced the misclassification and misinterpretation of the subgroup meta-analysis. Even though we successfully revealed the global prevalence of MRSA in dairy cattle farms, other knowledge such as the risk factors associated with the presence of MRSA and the antimicrobial use in dairy cattle farms was not described. This gap in knowledge is critical in controlling and monitoring MRSA in dairy cattle farms and needed to be further investigated in a future study.

## Conclusion

The global pooled prevalence of MRSA in dairy cattle farms has been minimal yet consistent over time. The pooled prevalence of MRSA in dairy cattle farms was the highest in Asia, followed by Africa and Europe. Cattle milk samples were found to harbor a higher prevalence of MRSA than other sample types. Therefore, following the results of this study, we recommend that appropriate levels of barn sanitation, personnel sanitation while handling animals and animal products, implementation of a continuous surveillance and monitoring program for evaluating animal health, and monitoring of antimicrobial resistance patterns at the farm level, be employed to control the spread of MRSA in dairy cattle farms.

## Data availability statement

The original contributions presented in the study are included in the article/Supplementary material, further inquiries can be directed to the corresponding author.

## Author contributions

SB and NA designed this study. SB, SK, and NA performed the systematic review and meta-analysis. SB and SK prepared and revised the manuscript accordingly. All authors contributed to the article and approved the submitted version.

## Funding

This study was funded by the Center of Excellence in Veterinary Public Health, Faculty of Veterinary Medicine, and CMU Presidential scholarship, Chiang Mai University.

## Conflict of interest

The authors declare that the research was conducted in the absence of any commercial or financial relationships that could be construed as a potential conflict of interest.

## Publisher's note

All claims expressed in this article are solely those of the authors and do not necessarily represent those of their affiliated organizations, or those of the publisher, the editors and the reviewers. Any product that may be evaluated in this article, or claim that may be made by its manufacturer, is not guaranteed or endorsed by the publisher.
