# Supplementary material for: Prevalence of methicillin-resistant Staphylococcus aureus in dairy farms: A systematic review and meta-analysis
Source: Front Vet Sci. 2022 Dec 6;9:947154. doi: 10.3389/fvets.2022.947154 (PMC9763730; doi:10.3389/fvets.2022.947154)
Supplement: Supplementary file 1 [file Table_1.DOCX]

Table S1. Characteristics of included studies for the meta-analysis

| Authors | Year | Country | Sample size | Sample source | No. of *S. aureus* | No. of MRSA | Detection method | Quality score |
| --- | --- | --- | --- | --- | --- | --- | --- | --- |
| Lee et al. (27) | 2003 | Korea | 966 | cattle milk, feces, feed | 269 | 12 | *mecA* | 10 |
| Moon et al. (28) | 2007 | Korea | 14,688 | cattle milk | 835 | 13 | *mecA* | 10 |
| Virgin et al. (29) | 2009 | USA | 542 | cattle milk | 190 | 7 | *mecA* | 7 |
| Kumar et al. (30) | 2010 | India | 185 | cattle milk | 128 | 10 | *mecA* | 7 |
| Asfour and Darwish (31) | 2011 | Egypt | 223 | cattle milk | 33 | 5 | *mecA* | 8 |
| Kumar et al. (32) | 2011 | India | 195 | cattle milk | 107 | 10 | *mecA* | 5 |
| Nithin Pravu et al. (33) | 2012 | India | 150 | cattle milk | 35 | 8 | *mecA* | 8 |
| Pehlivanoglu and Yardimci (34) | 2012 | Turkey | 306 | cattle milk | 65 | 12 | *mecA* | 6 |
| Matyi et al. (35) | 2013 | USA | 133 | cattle milk | 40 | 7 | *mecA* | 6 |
| Vandendriessche et al. (36) | 2013 | Germany | 113 | cattle nasal, human nasal | NR | 1 | *mecC* | 7 |
| Chandrasekaran et al. (37) | 2014 | India | 401 | cattle milk | 162 | 12 | *mecA* | 8 |
| Havaei et al. (38) | 2014 | Iran | 450 | cattle milk | 54 | 10 | *mecA* | 5 |
| Nemeghaire et al. (39) | 2014 | Germany | 141 | cattle nasal | NR | 14 | *mecA* | 6 |
| Paterson et al. (40) | 2014 | Great Britain | 1,090 | cattle milk | NR | 11 | *mecC* and *mecA* | 10 |
| Pu et al. (41) | 2014 | China | 450 | cattle milk | 103 | 49 | *mecA* | 10 |
| Schlotter et al. (42) | 2014 | Germany | 241 | cattle milk, cattle nasal, mammary skin, teat lesion, ankle, vagina, milk holder, leaked milk on floor, human hand and nasal | 38 | 18 | *mecC* and *mecA* | 8 |
| Visciano et al. (43) | 2014 | Italy | 120 | cattle milk, human and environment | 42 | 1 | *mecA* | 9 |
| Wang et al. (44) | 2014 | China | 353 | cattle milk | 53 | 0 | *mecA* | 9 |
| Zhao et al. (45) | 2014 | China | 260 | cattle uterine secretion | 53 | 0 | *mecA* | 10 |
| Da Costa Krewer et al. (46) | 2015 | Brazil | 2,064 | cattle milk | 126 | 0 | *mecA* | 5 |
| El-Ashker et al. (47) | 2015 | Egypt | 800 | cattle milk | 146 | 8 | *mecA* | 10 |
| Rahimi et al. (48) | 2015 | Iran | 79 | cattle nasal | 4 | 0 | *mecA* | 9 |
| Riva et al. (49) | 2015 | Italy | 282 | cattle milk | 30 | 5 | *mecA* | 8 |
| Weiner et al. (50) | 2015 | Poland | 650 | cattle milk | 45 | 45 | *mecA* | 7 |
| Akkou et al. (51) | 2016 | Africa | 347 | cattle milk, human nasal | 114 | 4 | *mecA* | 8 |
| Bau et al. (52) | 2016 | China | 121 | cattle milk | 52 | 5 | *mecA* | 9 |
| Cortimiglia et al. (53) | 2016 | Italy | 844 | cattle milk | 398 | 32 | *mecA* | 8 |
| Ganai et al. (54) | 2016 | India | 25 | cattle milk | 15 | 8 | *mecA* | 5 |
| Igbinosa et al. (55) | 2016 | Nigeria | 283 | cattle milk, cattle nasal | 30 | 30 | *mecA* | 10 |
| Mausam et al. (56) | 2016 | India | 150 | cattle milk | 85 | 44 | *mecA* | 7 |
| Mistry et al. (57) | 2016 | India | 167 | cattle milk | 39 | 19 | *mecA* | 5 |
| Oliveiria et al. (58) | 2016 | Brazil | 572 | cattle milk, human nasal | 70 | 22 | *mecA* | 9 |
| Parisi et al. (19) | 2016 | Italy | 486 | cattle milk | NR | 12 | *mecA* | 8 |
| Song et al. (59) | 2016 | Korea | 649 | cattle milk | 165 | 23 | *mecA* | 10 |
| Zhang et al. (60) | 2016 | China | 200 | cattle milk | 58 | 11 | *mecA* | 9 |

Table S1. Characteristics of included studies for the meta-analysis (continued)

| Authors | Year | Country | Sample size | Sample source | No. of S. aureus | No. of MRSA | *Detection method* | Quality score |
| --- | --- | --- | --- | --- | --- | --- | --- | --- |
| Aqib et al. (61) | 2017 | Pakistan | 450 | cattle milk | NR | 135 | *mecA* | 9 |
| Awad et al. (62) | 2017 | Egypt | 200 | cattle milk | 84 | 42 | *mecA* | 8 |
| Ektik et al. (63) | 2017 | Turkey | 50 | cattle milk | 17 | 0 | *mecA* | 7 |
| Guimarães et al. (64) | 2017 | Brazil | 115 | cattle milk | 60 | 29 | *mecA* | 6 |
| Ismail (65) | 2017 | Jordan | 188 | cattle milk | 30 | 7 | *mecA* | 8 |
| Klimešová et al. (66) | 2017 | Czech Republic | 114 | cattle milk, cattle nasal, cattle rectal swab, environment, human nasal and excrement | 1 | 0 | *mecA* | 7 |
| Kulangara et al. (67) | 2017 | India | 157 | cattle milk | 20 | 19 | *mecA* | 7 |
| Leigue et al. (68) | 2017 | Brazil | 150 | cattle milk, human nasal | 5 | 0 | *mecA* | 9 |
| Liu et al. (69) | 2017 | China | 195 | cattle milk | 54 | 0 | *mecA* | 6 |
| Sharma et al. (70) | 2017 | India | 368 | cattle milk | 73 | 15 | *mecA* | 10 |
| Gezgen and Seker (71) | 2018 | Turkey | 972 | cattle milk | 60 | 0 | *mecA* | 8 |
| Hamed (72) | 2018 | Egypt | 76 | cattle milk | 12 | 6 | *mecA* | 7 |
| Hoque et al. (73) | 2018 | Bangladesh | 200 | cattle milk | 145 | 29 | *mecA* | 10 |
| Khemiri et al. (74) | 2018 | Tunisia | 141 | cattle milk, cattle nasal | 27 | 1 | *mecA* | 6 |
| Klibi et al. (75) | 2018 | Tunisia | 300 | cattle milk | 15 | 3 | *mecA* | 8 |
| Liu et al. (76) | 2018 | China | 350 | cattle milk | 80 | 1 | *mecA* | 8 |
| Papadopoulos et al. (77) | 2018 | Greece | 45 | cattle milk, cattle nasal | 23 | 1 | *mecA* | 7 |
| Srednik et al. (78) | 2018 | Argentina | 829 | cattle milk | 229 | 0 | *mecA* | 9 |
| Tenhagen et al. (79) | 2018 | Germany | 675 | cattle milk | NR | 41 | *mecA* | 6 |
| Wang et al. (80) | 2018 | China | 195 | cattle milk | 90 | 1 | *mecA* | 9 |
| Yi et al. (81) | 2018 | China | 572 | cattle milk | 56 | 56 | *mecA* | 10 |
| Alnakip et al. (82) | 2019 | Spain | 100 | cattle milk | 5 | 0 | *mecA* | 4 |
| Amandeep et al. (83) | 2019 | India | 204 | cattle milk | 64 | 3 | *mecA* | 6 |
| Dan et al. (84) | 2019 | China | 337 | cattle milk, endometritis swab | 155 | 22 | *mecA* | 9 |
| Käppeli et al. (85) | 2019 | Switzerland | 58 | cattle milk | 58 | 0 | *mecA* | 8 |
| Khan et al. (86) | 2019 | Pakistan | 74 | cattle milk | NR | 40 | *mecA* | 9 |
| Mushtaq et al. (87) | 2019 | India | 160 | cattle milk | 52 | 7 | *mecA* | 6 |
| Oreiby et al. (88) | 2019 | Egypt | 68 | cattle milk | 17 | 5 | *mecA* | 6 |
| Rossi et al. (89) | 2019 | Brazil | 665 | cattle milk | 116 | 4 | *mecA* | 9 |
| Shah et al. (90) | 2019 | India | 150 | cattle milk | 80 | 20 | *mecA* | 6 |
| Tegegne et al. (91) | 2019 | Czech Republic | 29 | cattle milk | NR | 11 | *mecA* | 10 |
| Aklilu and Chia (92) | 2020 | Malaysia | 95 | cattle milk, cattle nasal | 44 | 17 | *mecA* | 9 |
| Algammal et al. (93) | 2020 | Egypt | 146 | cattle milk | 53 | 20 | *mecA* | 10 |
| Antok et al. (94) | 2020 | Rwanda | 303 | cattle milk | 43 | 0 | *mecA* | 10 |
| Dastmalchi Saei and Panahi (95) | 2020 | Iran | 319 | cattle milk, cattle nasal | 32 | 2 | *mecA* | 10 |
| El-Ashker et al. (96) | 2020 | Egypt | 550 | cattle milk | 42 | 12 | *mecA* | 8 |
| Ewida and AI-Hosary (97) | 2020 | Egypt | 60 | cattle milk | 48 | 0 | *mecA* | 8 |
| Girmay et al. (98) | 2020 | Ethopia | 64 | cattle milk | 21 | 5 | *mecA* | 10 |
|  |  |  |  |  |  |  |  |  |

Table S1. Characteristics of included studies for the meta-analysis (continued)

| Authors | Year | Country | Sample size | Sample source | No. of S. aureus | No. of MRSA | *Detection method* | Quality score |
| --- | --- | --- | --- | --- | --- | --- | --- | --- |
| Kalayu et al. (99) | 2020 | Ethopia | 456 | cattle milk, human nasal | 70 | 1 | *mecA* | 10 |
| Keyvan et al. (100) | 2020 | Turkey | 120 | cattle milk | 53 | 40 | *mecA* | 6 |
| Liu et al. (101) | 2020 | China | 624 | cattle milk | 62 | 8 | *mecA* | 9 |
| Ramandinianto et al. (102) | 2020 | Indonesia | 150 | cattle milk | 92 | 2 | *mecA* | 10 |
| Ren et al. (103) | 2020 | China | 84 | cattle milk | 65 | 0 | *mecA* | 9 |
| Silva et al. (104) | 2020 | Brazil | 432 | cattle milk | 27 | 0 | *mecA* | 9 |
| Yang et al. (105) | 2020 | China | 3,136 | cattle milk | 498 | 73 | *mecA* | 6 |
| Zayda et al. (106) | 2020 | Egypt | 225 | cattle milk, cheese | 25 | 15 | *mecA* | 6 |
| Abdeen et al. (107) | 2021 | Egypt | 140 | cattle milk | 43 | 33 | *mecA* | 6 |
| Chen et al. (108) | 2021 | China | 466 | cattle milk | 125 | 9 | *mecA* | 7 |
| Crespi et al. (109) | 2021 | Argentina | 148 | cattle milk | 31 | 1 | *mecA* | 7 |
| Duse et al. (110) | 2021 | Sweden | 779 | cattle milk | 227 | 0 | *mecA* | 8 |
| Gharghi et al. (111) | 2021 | Iran | 100 | cattle milk | 6 | 6 | *mecC* and *mecA* | 6 |
| Khazaie and Ahmadi (112) | 2021 | Iran | 283 | cattle milk | 96 | 11 | *mecA* | 7 |
| Lemma et al. (113) | 2021 | Ethopia | 175 | cattle milk | 43 | 1 | *mecA* | 10 |
| Nhatsave et al. (114) | 2021 | Mozambique | 143 | cattle milk | 58 | 2 | *mecA* | 10 |
| Patel et al. (115) | 2021 | USA | 365 | cattle milk | 124 | 1 | *mecA* | 6 |
| Qolbaini et al. (116) | 2021 | Indonesia | 86 | cattle milk | 49 | 9 | *mecA* | 10 |
| Shrestha et al. (117) | 2021 | Nepal | 191 | cattle milk | 29 | 2 | *mecA* | 10 |
| Tegegne et al. (118) | 2021 | Ethopia | 265 | cattle milk | 100 | 12 | *mecA* | 10 |
| Zhao et al. (119) | 2021 | China | 418 | cattle milk | 121 | 3 | *mecA* | 10 |

NR: not reported

Table S2. Summary results of univariable meta-regression for the overall pooled prevalence of MRSA in dairy cattle farms

| Variables | Coefficient  (95% confidence interval) | Standard error | *p*-value |
| --- | --- | --- | --- |
| Publication year |  |  |  |
| After 2018 (ref) | - |  |  |
| 2015 to 2018 | 0.0037 (-0.0620 to 0.0693) | 0.0335 | 0.9122 |
| Before 2015 | -0.0394 (-0.1181 to 0.0393) | 0.040 | 0.3260 |
| Continent |  |  |  |
| Africa (ref) | - |  |  |
| Asia | 0.0209 (-0.0536 – 0.0953) | 0.0380 | 0.5832 |
| Europe | -0.0135 (-0.0997 – 0.0727) | 0.0440 | 0.7591 |
| North America | -0.0663 (-0.2470 – 0.1145) | 0.0922 | 0.4724 |
| South America | -0.0729 (-0.1866 – 0.0408) | 0.0580 | 0.2089 |
| Sample type |  |  |  |
| Cattle milk (ref) |  |  |  |
| Others | -0.0298 (-0.1080 – 0.0485) | 0.0399 | 0.4559 |

Table S3. Summary results of multivariable meta-regression for the overall pooled prevalence of MRSA in dairy cattle farms

| Variables | Coefficient  (95% confidence interval) | Standard error | *p*-value |
| --- | --- | --- | --- |
| Publication year |  |  |  |
| After 2018 (ref) | - |  |  |
| 2015 to 2018 | 0.0112 (-0.0543 to 0.0768) | 0.0355 | 0.7370 |
| Before 2015 | -0.0384 (-0.1234 – 0.0466) | 0.0434 | 0.3760 |
| Continent |  |  |  |
| Africa (ref) | - |  |  |
| Asia | 0.0268 (-0.0488 to 0.1024) | 0.0386 | 0.4878 |
| Europe | 0.0017 (-0.0889 to 0.0922) | 0.0462 | 0.9715 |
| North America | -0.0414 (-0.2285 to 0.1457) | 0.0955 | 0.6644 |
| South America | -0.0785 (-0.1926 to 0.0356) | 0.0582 | 0.1774 |
| Sample type |  |  |  |
| Cattle milk (ref) | - |  |  |
| Others | -0.0183 (-0.0982 to 0.0617) | 0.0408 | 0.6541 |
